# Women’s perception and risk factors for delayed initiation of breastfeeding in Arba Minch Zuria, Southern Ethiopia

**DOI:** 10.1186/1746-4358-9-8

**Published:** 2014-06-13

**Authors:** Dessalegn Tamiru Adugna

**Affiliations:** 1 Nutrition Unit, Public Health Department, Arba Minch University, Arba Minch, Ethiopia

**Keywords:** Breastfeeding initiation, Delayed initiation, Ethiopia

## Abstract

**Background:**

Breastfeeding is one of the components of Primary Health Care in Ethiopia. In Ethiopia a wide range of harmful infant feeding practices has been documented despite the implementation of infant and young child feeding guidelines. However, there is no well documented study of women’s perception of breastfeeding patterns and factors associated with delayed initiation of breastfeeding (with timely initiation of breastfeeding being within the first hour) in rural communities of Arba Minch Zuria.

**Methods:**

A community-based cross-sectional study was carried out in Arba Minch Zuria from January to February, 2012. Quantitative data were collected from a sample of 383 respondents supplemented by qualitative data generated using in-depth interviews of 10 key informants. A multivariate logistic regression analysis was used to identify the predictors of delayed initiation of breastfeeding practices. Qualitative data were analyzed using thematic frameworks.

**Results:**

In the rural communities of Arba Minch Zuria almost all mothers (98.2%) have ever breastfed their children. More than three-fourth (89%) of mothers provided colostrum to their infants while others discarded the first milk until the white milk was produced. A large number of mothers (42.8%) started breastfeeding one hour after childbirth. Delayed initiation of breastfeeding was positively associated with lack of maternal education (AOR 1.91; 95% CI 1.02, 3.44). Maternal knowledge about the duration of exclusive breastfeeding (AOR 0.39; 95% CI 0.15, 0.93), attending a primary health education (AOR 0.74; 95% 0.15, 0.98) and health personnel support for women at delivery time (AOR 0.52; 95% CI 0.21, 0.58) were inversely associated with delayed initiation of breastfeeding practices.

**Conclusions:**

A large number of mothers (42.8%) were short of the national and global recommendations about breastfeeding initiation. Therefore, sustained health and community based nutritional education is recommended for pregnant and lactating mothers to promote optimal breastfeeding for the initiation of breastfeeding practices using health extension workers and local community resource people as key actors.

## Background

Sub-optimal child feeding practices and dietary inadequacy is the major public health problem resulting in serious social and economic consequences in the world [1-6]. Studies have shown that the life of 1.5 million infants and young children could be saved by following optimal feeding practices. According to the World Health Organization (WHO) report, only 35% of mothers exclusively breastfed their children for six months and large proportions of infants do not receive timely and adequate complementary feeding globally. Despite its benefits, timely initiation of breastfeeding within the first hour is often delayed by due to traditional practices like prelacteal feeding and social beliefs in developing countries [5-10].

In developing countries like Ethiopia early and abrupt cessation of breastfeeding, delayed initiation of breastfeeding, introduction of dirty and unsound artificial feeding of infants with very dilute milk products is common [1,5,11]. As a result, infants and young children are exposed to malnutrition and infectious disease as their body nutrient stores are not well developed [3,4,12].

Breastfeeding is the natural, biological way of providing children with the nutrients required for healthy growth and development. The WHO recommends early initiation of breastfeeding as it provides many nutritional, immunological and psychosocial benefits, including protection of the infant against infectious diseases and enhanced maternal infant bonding [5,6,13]. Breastfeeding initiation within the first hour of childbirth is the easiest and most successful when a mother is physically and psychologically prepared for breastfeeding and when she is supported and informed about the advantages of breastfeeding practices [6,8,13]. Early initiation of breastfeeding also confirms that a newborn receives colostrum. Colostrum is thick yellowish breast milk, highly nutritious, digestible and it is the most immunologically protective secretion of the mammary gland during lactogenesis. In many developing countries, some women discard colostrum on the basis of traditional or cultural beliefs seeing it as harmful to the infant’s health [6-11].

Breastfeeding is nearly universal in Ethiopia. However, large proportions of mothers, both urban and rural, do not practice optimal infant feeding behaviors [12,13]. In Ethiopia there is high infant and young child mortality due to sub-optimal feeding practices and infectious disease. The majority of these deaths are attributable to sub-optimal breastfeeding practices. This has great impact on child growth and developments where the accessibility of basic needs and health services are not adequately available [3,12,13].

Establishing appropriate breastfeeding in the first day is critical to the health of the newborn infant and to breastfeeding success. However, the timing of initiation of breastfeeding is variable in length and is influenced by different sociocultural factors, economic and demographic factors [6,8,9]. Additionally, mothers in difficult situations require special attention and practical support to be able to feed their children appropriately as the probability of breastfeeding decreases and there may be an increase of artificial feeding and inappropriate complementary feeding practices [10,11,14].

In rural areas of Ethiopia, 49% of mothers started breastfeeding after one hour of birth. The majority of women delayed initiation of breastfeeding due to lack of time and introduction of prelacteal feeds. In Ethiopia 27% of infants are given prelacteal feeds like butter, rue and fenugreek prior to breastfeeding [3,9,12]. Although breastfeeding is one of the components of Primary Health Care in Ethiopia, a wide range of harmful infant feeding practices are documented, even after implementation of infant and young child feeding recommendations. However, there is no well documented study of women’s perception of breastfeeding patterns and factors associated with delayed initiation of breastfeeding in the study area. Therefore the aim of this study was to assess women’s perception and risk factors for delayed initiation of breastfeeding in rural communities of Arba Minch Zuria, Gamo Gofa Zone.

## Methods

### Study setting and sampling

A community based survey was carried out in Arba Minch Zuria from January to February, 2012. Arba Minch Zuria is a part of the Gamo Gofa zone, which is located in the Southern part of Ethiopia. From 31 kebeles (administrative units) of Arba Minch Zuria, 9 kebeles (administrative units) were randomly selected. Gamo, Wolayita, Zeyse, Amhara and Oromo ethnic groups made up to 97% of the Arba Minch Zuria population. Based on figures published by the Central Statistical Agency in 2007, this district has an estimated total population of 165,680 [15].

A sample size of 384 breastfeeding mothers was calculated using a single population proportion formula with a 5% margin of error, 95% confidence interval and 50% estimated prevalence of delayed initiation of breastfeeding in the study area. The inclusion criteria were women who had children under two years and permanent resident of selected kebeles (administrative units). Mother-child pairs from each selected kebele (administrative unit) were randomly selected using a simple random sampling technique from the list women who had children within each kebele (administrative unit). Ten key informant mothers were purposely selected based on their role in the community for in-depth interviews to capture their first-hand knowledge about child feeding practices.

### Measurements

The study used structured and in-depth interview questionnaires. The survey included questions concerning sociodemographic variables, maternal and child characteristics, child feeding practices and environmental health. Furthermore, women’s knowledge of the benefits of breast milk, and sociocultural influences of child feeding were also included in the questionnaire. The aim of in-depth interviews with breastfeeding mothers and traditional birth attendants were to generate women’s perception, experiences and knowledge of breastfeeding. In-depth interviews were conducted for approximately one hour at the study participant’s home by audio tape recording and transcribed verbatim.

Questionnaires were prepared in English and translated to Amharic by language experts. Finally, it was retranslated back to English by a person who can speak both languages. After the recruitment of data collectors questionnaires were pre-tested for its understandability and time allocation. Based on a pretest additional adjustment was made in terminologies and formatting questionnaires.

### Data analysis

Data were analyzed using SPSS (SPSS Inc. version 16.0, Chicago, Illinois). The data were checked for missing values and outliers. Bivariate associations between predictors and delayed initiation of breastfeeding were described to determine the strength of association using odds ratios and 95% of confidence intervals. To identify the predictors of delayed initiation of breastfeeding, only variables that were significantly associated on bivariate analyses were entered into the multivariable logistic regression model with delayed initiation of breastfeeding as the dependent variable. In this study, multicollinearity among independent variables was detected using the standard errors for regression coefficients. The standard errors < 2.0, as a familiar cutoff value, showed that there was no multicollinearity [16]. All tests were two-sided and p < 0.05 was considered statistically significant.

Qualitative data were analyzed based on the response from respondents. In-depth interview data were compared and discussed in detail. Qualitative data were analyzed by open coding, which involves sorting the data into analytical categories by breaking down, examining, comparing and categorizing data. Finally the categories of data were compared and contrasted to generate themes from the analysis. On completion of the research, qualitative findings were presented in triangulation with the quantitative findings.

### Ethical considerations

The study was conducted after getting official permission from an ethical clearance committee of Arba Minch University and Arba Minch Zuria Woreda administration. Prior to the first interview each respondent was informed about objective of the study and privacy during the interview. Informed verbal consent was obtained from each study participant.

## Results

Of the intended 384 participants, 383 (99.7%) responded. The mean (±standard deviation) age of the mothers was 25.08 years (±6.65). More than two-third of families (68.4%) had a family size greater than 5 people. The majority of mothers (61.4%) did not attend any types of formal education (Table 1).

**Table 1:** Sociodemographic characteristics of respondents in Arba Minch Zuria, 2012 (n = 383)

**Variable**	**Frequency**	**Percent**
Marital status		
Married	376	98.2
Unmarried	7	1.8
Family size		
< 5	121	31.6
≥ 5	262	68.4
Maternal age (in years)		
> 20	83	21.67
20–24	230	60.05
≥ 25	70	18.28
Attended antenatal care		
Yes	319	83.3
No	64	16.7
Utilization of family planning		
Yes	237	61.9
No	146	38.1
Delivery assistant		
Traditional birth attendants	197	51.4
Health workers	41	10.7
Relatives	145	37.9
Child age (in months)		
0–6	120	31.33
7–12	170	44.39
13–24	93	24.28
Delivery type		
Caesarean section	6	1.6
Vaginal delivery	377	98.4
Place of delivery		
Home	370	96.6
Health institution	13	3.4
Maternal education		
No education	235	61.4
Reading and writing	79	20.6
Elementary and above	69	19
Ethnicity		
Gamo	299	78.0
Wolaita	19	5.0
Other*	65	17.0
Farm land owners		
No	94	75.5
Yes	289	24.5
Religion		
Protestant	234	61.1
Orthodox Christian	138	36.0
Others**	11	2.9

*Zeyse, Amhara, Ganta, Gofa; **Muslim, Traditional.

Almost all mothers (98.2%) had ever breastfed their children in the rural communities of Arba Minch Zuria. However, a large number of mothers (42.8%) started breastfeeding after one hour of childbirth. More than three-fourth (89%) of mothers provided colostrum to their infants while others discarded the first milk until the white milk was produced (Table 2).

**Table 2:** Breastfeeding pattern in Arba Minch Zuria, 2012 (n = 383)

**Variables**	**Frequency**	**Percent**
Ever breastfed		
Yes	376	98.2
No	7	1.8
Breastfeeding initiation		
Within one hour	219	57.2
After one hour	164	42.8
Discarded colostrum		
Yes	42	11
No	341	89
Breastfeeding at night		
Yes	372	97.1
No	11	2.9
Breastfeeding frequency		
0	7	1.8
1–8	207	54.1
≥ 8	169	44.1
Duration of EBF in month		
< 1	32	8.4
2–3	28	7.3
4-5	60	15.7
6	213	55.6
7 and above	50	13.1
Prelactael feeding		
Yes	34	8.9
No	349	91.1
Type of pre-lacteal food (n = 34)		
Cow’s milk	8	23.5
Water	14	41.2
Other	12	35.3
Giving additional food		
< 6 month	120	31.3
≥ 6 month	263	68.7
Feeding utensil		
Bottle	8	2.1
Cup	136	35.5
Spoon	63	16.4
No instrument	176	46

Two-hundred thirteen mothers (55.6%) exclusively breastfed their children for six months. However, 120 (31.33%) mothers gave additional food before six months. More than one-third of mothers (35.50%) used cup to feed their infants. Among prelacteal feeds, 14 (41.18%) of mothers provided water as they believed that it used to remove waste from the infant’s stomach (Table 2).

One-hundred eighty-three mothers (47.78%) reported they didn’t have information about the importance of colostrum. Some women (8.4%) gave cow’s milk instead of initiating breastfeeding since they considered colostrum as out of date milk (Table 3).

**Table 3:** Maternal attitude and knowledge about colostrum and breastfeeding initiation in Arba Minch Zuria, 2012

**Knowledge and attitude**	**Frequency**	**Percent**
**Knowledge**		
Know the importance of colostrum	142	27.1
Has no concept about colostrum	183	47.8
Know breastfeeding initiation within 1 hour	150	39.2
**Attitude**		
Believe breast milk is good for the health of a child	350	91.4
Accept discarding colostrum as a culture	4	1.0
Believe colostrum causes disease	33	8.6
Perceive discarding colostrum as a norm	4	1.0
Favor cow’s milk instead of discarded colostrum	32	8.4
Believe prelacteal feeding is good for infants	30	7.8

Delayed initiation of breastfeeding was significantly associated with lack of maternal education, knowledge gap about the duration of exclusive breastfeeding, home delivery without the help of health professionals and deficits of primary health education about breastfeeding initiation (Table 4).

**Table 4:** Factors associated with delayed initiation of breastfeeding in Arba Minch Zuria, 2012 (n = 383)

**Variable**	**Delayed initiation of breastfeeding**			
	**Yes**	**No**	**Crude Odds Ratio (95% CI)**	**Adjusted Odds Ratio (95% CI)**
Maternal age				
15–19	33	50	0.78 (0.41, 1.49)	_
20-24	99	131	0.90 (0.52, 1.54)	
≥ 25	32	38	1	
Maternal education				
No education	112	123	1.95 (1.103, 3.43)*	1.91 (1.02, 3.44)*
Reading and writing	30	49	1.308 (0.66, 2.58)	1.36 (0.67, 2.78)
Elementary and above	22	47	1	1
Number of children < 5 years				
1	119	149	1.24 (0.80, 1.94)	_
≥ 1	45	70	1	
Antenatal care follow up				
Yes	140	179	1.30 (0.75, 2.26)	_
No	24	40	1	
Birth place				
Home	158	212	0.87 (0.29, 2.64)	_
Health institution	6	7	1	
Delivery assistant				
Traditional Birth Attendants	83	114	0.87 (0.57, 1.34)	0.85 (0.53, 1.31)
Health workers	15	26	0.69 (0.34, 1.41)	0.52 (0.21, 0.58)*
Relatives	66	79	1	1
EBF knowledge to 6 months				
Yes	148	211	0.35 (0.15, 0.84)**	0.39 (0.15, 0.93)*
No	16	8	1	1
Radio owners				
Yes	78	106	0.97 (0.65, 1.45)	_
No	86	113	1	
Attending health education				
Yes	107	159	0.71 (0.46, 1.10)	0.74 (0.15, 0.98)**
No	57	60	1	1
Pre-lactateal feeding				
Yes	17	17	1.37 (0.68, 2.78)	_
No	147	202	1	
Discarded colostrum				
Yes	21	21	1.31 (0.73, 2.63)	_
No	143	198	1	
Total number of births				
1–2	62	84	0.98 (0.64, 1.48)	_
> 2	102	135	1	

**Significant at 0.01, *Significant at 0.05.

Maternal education status was significantly associated with delayed initiation of breastfeeding. Women who did not attend any formal education were nearly twice as likely not to initiate breastfeeding in a timely manner (AOR 1.91; 1.02, 3.44) as compared to those women who attended primary school and higher education. Similarly, mothers who had knowledge about the duration of exclusive breastfeeding were 61% less likely to delay breastfeeding initiation (AOR 0.39; 0.15, 0.93) than mothers who had no knowledge about the duration of exclusive breastfeeding.

The women whose delivery was attended by skilled health professionals were 48% less likely to delay breastfeeding initiation (AOR 0.52; 0.21, 0.58) when compared with those who were attended by their relatives.

Community-based health education given at different occasion targeted to promote optimal breastfeeding was positively associated with timely initiation of breastfeeding practices. Women who attended health education about breastfeeding practices were 26% more likely to initiate timely breastfeeding (AOR 0.74; 0.15, 0.98) than who did not attend.

Similar to quantitative findings, qualitative data also showed that some women discarded colostrum as they perceived that it causes disease. Instead of colostrum, they provided butter, fenugreek and water for infants. They considered colostrum as expired breast milk and substance which can endanger child health. A twenty-nine year old lady farmer who had a 7 month old infant said:

“Colostrum has a yellowish color and it is an expired substance; It can cause colic to the newborn baby.”

However, some women had basic concepts about the dietary importance of colostrum for newborn infants. They reported that first milk is used as preventive medicine to fight against disease. A thirty-two year old traditional birth attendant woman who had a 2 month old child said:

“Previously colostrum was considered as an expired substance. However, at this moment majority of mothers were well informed about the dietary importance of colostrum. They considered colostrum as the first stage of immunization.”

Findings of in-depth interview also showed that mothers who had information about the dietary importance of breast milk were initiating breastfeeding within the first hour of childbirth. A twenty-one year old merchant woman who had a 2 month old infant said:

“Unlike to our descendants, this generation is well informed about the nutritional value of colostrum and early initiation of breastfeeding. However, in the earlier time butter, water and fenugreek will be given as a prelacteal feeds.”

Some mothers also reported that nutritional education through the media and by health workers had great input in optimal infant and child feeding practices. A thirty year old farmer woman who had 11 month old infant said:

“Now we have information about the impacts of traditional malpractices and the dietary importance of timely initiation of breastfeeding as there is routine health and nutritional education about this issue.”

Similarly, mothers who were counseled about the advantages of early initiation of breastfeeding practices at health post have provided breast milk within the first hour of child birth. A thirty-three year old housewife with a 3 month old infant said:

“… I gave breast milk within one hour of child birth since I had been already informed about its advantage.”

## Discussion

Breastfeeding is a common practice and considered as a natural gift in Ethiopia [3,13]. Despite national attempts to optimize breastfeeding practices, 43% of mothers in Arba Minch Zuria started breastfeeding after one hour of childbirth which is relatively low when compared with 2011 Ethiopian demographic and health survey (EDHS), which was 52%. Findings of qualitative data showed that social beliefs and traditional malpractices had a significant role in delayed initiation of breastfeeding [3,11].

The findings of this study showed that 56% of mothers provided only breast milk for their children to six months. Despite national and international recommendations, a large number of women (31%) gave additional foods to their infants before six months since they believed that breast milk alone cannot meet the nutritional need of their infants. This might be due to inadequate availability of information about the adequacy of breast milk for infants for the first six months of life. The majority of women use a cup (36%), which is more preferable to limit the cross transmission of food borne disease to feed their infants. Ethiopian infant and young child feeding guidelines similarly encourage women to use a cup rather than bottle due problems of hygiene [3,12,13].

Provision of colostrum to newborn infants was common practice in rural communities of Arba Minch Zuria. Nevertheless, a large number of mothers (48%) had no information about the dietary importance of colostrum though majority of them did not discard it. Some women considered colostrum as expired milk and gave prelacteal feeds instead and discarded the colostrum. This finding is consistent with the study done in the Gambia and India in which some women discarded colostrum due to lack of knowledge about dietary importance of colostrum and traditional beliefs [14,17]. An in-depth interview with some women showed that perception about colostrum was changed and the majority of women were providing first milk to infants after receiving occasional advice about optimal breastfeeding practices by health workers for the pregnant and lactating mothers.

Among those women who gave prelacteal feeds, 41% of women considered provision of water as a means of cleaning the infant’s stomach. This might be due to traditional beliefs and knowledge gap on the dietary value of breast milk. The studies done in India and Uganda also showed that prelacteal feeds were perceived as preventive medicines to fight against disease [18,19].

This study indicated that lack of maternal education is one of the predisposing factors to delayed initiation of breastfeeding practices. This might be due to lack of basic information about the dietary importance of the first milk and long life health impacts of early initiation of breastfeeding. Similar evidence from in-depth interviews showed that attending health education demonstrates a great role in the promotion of optimal breastfeeding practices. Similar studies have also shown that maternal knowledge about dietary importance of breastfeeding is a predetermining factor in enhancing optimal child feeding practices [20].

Maternal knowledge about the duration of exclusive breastfeeding for six months has a significant role in the promotion of early initiation of breastfeeding. This indicates educational intervention has a significant contribution in enhancing optimal child feeding practices. In-depth interviews with some mothers also showed that extensive education on optimal breastfeeding had a significant contribution in promoting appropriate breastfeeding practices. Studies also suggested that community based education has a significant role to change child feeding malpractices and enhance optimal breastfeeding practices [9,21,22].

Maternal health services have a potential role in the enhancement of optimal breastfeeding practices. Women who gave birth with the aid of health professionals were less likely to delay the initiation of breastfeeding. This finding is consistent with the study done in a Bale Goba district where timely initiation of breastfeeding was significantly associated with institutional delivery. This might be due to counseling and support given on breastfeeding practices during delivery time [23].

The findings of this study suggested that health education has a significant contribution to the enhancement of timely initiation of breastfeeding. Women who attended health education given by health extension workers were more likely to initiate early breastfeeding compared to who did not attend health education. Qualitative findings also showed that health education plays a great role in enhancing the initiation of breastfeeding. Studies also showed that nutritional education makes a significant contribution in the promotion of optimal child feeding practices and reduction of exposure to infectious disease [6,8,23].

The findings of this study can be used as baseline data for policy makers for the formulation of child feeding strategies and achievement of millennium development goals to reduce child mortality. However, there are some limitations due to the cross-sectional nature of this study which limits to set a causal-effect relationship between dependent and independent variables. There might also be recall bias on breastfeeding practices as the mothers may forget when they initiated breastfeeding. However, training was given to data collectors on how to probe mothers and relate breastfeeding practices to local events to minimize recall bias.

## Conclusions

The findings of this study showed that a large number of mothers (43%) were not initiating breastfeeding in a timely manner (within 1 hour). This study revealed that lack of maternal education, knowledge gap about optimal breastfeeding practices and home delivery without the help of health professionals were the most important predictors of delayed of initiation breastfeeding practices. Policy makers and non-governmental organization should take into account the role of community based education on optimal breastfeeding and the importance of women’s education when designing intervention strategies and in promotion of strong community based networks using health extension workers and local community resource people as key players to encourage timely initiation of breastfeeding.

## Competing interests

The author declares that he has no competing interest.
